# Epidemiology of osteoporosis and fractures in ankylosing spondylitis

**DOI:** 10.1186/ar3724

**Published:** 2012-03-08

**Authors:** Philip N Sambrook

**Affiliations:** 1 Institute of Bone & Joint Research, University of Sydney, Sydney, Australia

## 

Osteoporosis and fractures, especially vertebral fractures, are a frequent complication of ankylosing spondylitis, even in the early stages of the disease. The risk of osteoporosis appears to be associated with elevated biochemical markers of bone turnover, increased pro-inflammatory cytokines, lower BMD, lower body weight and longer disease duration.

Using the WHO criteria (T score < -2.5), the prevalence of osteoporosis determined by BMD is about 29% in the spine and 12% in the hip in patients with ankylosing spondylitis, which compares with 2% and 1% respectively for controls. Although BMD is generally lower in patients with early disease, with progressive disease, the spine site becomes much less reliable due to the presence of syndesmophytes and periosteal new bone formation. In advanced cases, DXA becomes less reliable but using QCT, bone loss can be shown to continue within the vertebra and hips as well as an increase in cortical BMD and width.

The epidemiology of fractures in ankylosing spondylitis remains unclear although vertebral fractures have been most studied. The risk of clinical vertebral fractures is significantly increased (OR approximately 7.7 95% CI 4.3-12.6) and the cumulative incidence of clinical vertebral fractures is higher in men (OR 10.7 versus 4.2 in women) and increased especially during the first 5 years of the disease. The prevalence and incidence of non-vertebral fractures has been less well studied but in most reports appears to be about the same as in the control population.

Managing skeletal complications in anklosing spondylitis should include DXA of the spine and hip early in the disease. In more advanced disease, spinal DXA is not a useful predictor of fracture. In these circumstances, QCT should be considered. If a vertebral fracture is suspected, spinal imaging is required in order to avoid delays in diagnosis and therapy.

